# Protective Effect of DPPD on Mercury Chloride-Induced Hepatorenal Toxicity in Rats

**DOI:** 10.1155/2020/4127284

**Published:** 2020-07-15

**Authors:** Ahmed Nabil, Mohamed M. Elshemy, Medhat Asem, Heba F. Gomaa

**Affiliations:** ^1^Research Center for Functional Materials, National Institute for Materials Science (NIMS), 1-1 Namiki, Tsukuba, Ibaraki 305-0044, Japan; ^2^Biotechnology and Life Sciences Department, Faculty of Postgraduate Studies for Advanced Sciences (PSAS), Beni-Suef University, Beni-Suef, Egypt; ^3^Faculty of Science, Menoufia University, Menoufia, Egypt; ^4^Zoology Department, Faculty of Science, Ain-Shams University, Cairo, Egypt; ^5^Biology Department, Faculty of Sciences and Arts-Scientific Departments, Qassim University, Buraydah, Saudi Arabia

## Abstract

Mercury is a global environmental pollutant, accumulating mainly in the kidney and liver inducing hepatorenal toxicity, oxidative stress, and tissue damage. Oxidative stress is caused by an imbalance between free radicals' production and cellular antioxidant defense systems. In the present study, we investigated the effect of N N′-diphenyl-1, 4-phenylenediamine (DPPD) antioxidant activity against mercury chloride- (HgCl_2_-) induced renal and hepatic toxicity. Thirty adult female Sprague Dawley rats were divided into three equal groups: the first group was injected with saline only and served as a control, the second group was injected with HgCl_2_, and the third group received DPPD + HgCl_2_ rats injected with HgCl_2_ without treatment showing a significant increase in alkaline phosphatase (ALP), aspartate aminotransferase (AST), alanine aminotransferase (ALT), urea, creatinine, and uric acids compared to control. Moreover, the second group showed a significant reduction in the activity of the antioxidant enzymes (superoxide dismutase (SOD), catalase (CAT), and glutathione peroxidase (GSH)) in addition to a marked increase in the malondialdehyde (MDA) content, histopathological alterations, collagen deposition, CD8%, CD4%, and TGF-*β*% in kidney and liver tissues compared with the control group. Treatment with DPPD showed significant recovery (*p* ≤ 0.001) in all previous parameters and histopathological examination. In conclusion, we suggested that DPPD may have a promising antioxidant capacity, gives it the applicability to be used as a prophylactic agent against mercury-induced hepatorenal cytotoxicity in the future.

## 1. Introduction

Mercury is one of the most toxic metals responsible for environmental pollution [1]. Exposure to mercury in any of its forms in different ways such as water, air, soil, and food poses serious threats to our health and the environment [2]. Following exposure, mercury ions are taken up by and accumulate in numerous organs, including the brain, intestine, kidney, liver, and placenta [3]. Based on the available experimental data, it is a reasonable hypothesis that mercury toxicity involves oxidative stress, inflammation, and apoptosis [4]. HgCl_2_, as one of the most toxic salts of mercury, is metabolized primarily in the liver and, then, accumulated in the kidneys. Consequently, the liver and kidneys are considered the most affected organs [5]. HgCl_2_ demolishes free radical scavenging systems such as superoxide dismutase and catalase [6], as well as increase reactive species levels that lead to disturbance of the prooxidant-antioxidant balance system causing a condition of oxidative stress [7].

N N′-diphenyl-1, 4-phenylenediamine, a grey or dark grey powder, is used as an antioxidant in rubber and oils, especially for tires in industry due to its colour and stability [8]. DPPD is one of the most frequently used and potent antioxidants. It is effective at very low concentrations and believed to be rather selective [9]. DPPD, acting as an intracellular antioxidant, enlarges the pool size of lipid-soluble antioxidants, especially in the cytoplasmic membranes, and prevents the formation of lipid peroxides resulting in maintenance of the normal mitochondrial structure and enzyme activity [10]. The antioxidant activity of DPPD implemented by the donation of hydrogen to radical derivatives breaking the autocatalytic cycle protecting cells from oxidative stress [11] suppresses necrosis and decreases reactive oxygen species (ROS) formation [12] Also, DPPD inhibits lipid peroxidation and nephrotoxicity [13]. Thus, DPPD inhibits collagen deposition, dampens apoptosis, and prevents histopathological damages [14]. The present study reports the antifibrotic effect of DPPD against hepatorenal fibrosis induced by HgCl_2_ in rats.

## 2. Materials and Methods

### 2.1. Chemicals and Reagents

All chemicals and reagents were of the highest purity grade. DPPD (≥99.8%) and HgCl_2_ (≥99.5%) were obtained from Sigma-Aldrich Chemical Company (St. Louis, MO, USA). In addition to serum ALT, AST, and ALP activities, urea, uric acid, and creatinine levels were determined using colourimetric diagnostic kits (Biodiagnostic, Cairo, Egypt) according to the manufacturer's instructions. TGF-*β*%, CD4%, and CD8% were analyzed by using an Accuri C6 flow cytometer (BD Biosciences, San Jose, CA). Data were quantified with C Flow software (BD Accuri, San Jose, CA). The hydroxyproline content is measured by ELISA as an important index reflecting the degree of kidney and liver fibrosis.

### 2.2. Animals and Experimental Design

Rats were assigned to groups by using the Statistical Package of Social Science (SPSS) program for Windows (Standard version 21). Thirty female Sprague Dawley rats, weighing approximately 170–220 gm, were purchased from the Medical Experimental Research Center (MERC), Faculty of Medicine, Mansoura University, Mansoura, Egypt. The animals were kept in polypropylene cages under standard laboratory conditions of relative humidity (45 ± 5%) and temperature (25 ± 2°C) with 12 h light/dark cycle and provided with food pellets and tap water ad libitum. Principles of laboratory animals caring (NIH publication no. 85–23, that revised 1985) were followed. Ethical protocols for laboratory animal care and use were approved and followed under the supervision of Faculty of Postgraduate Studies for Advanced Sciences (PSAS), Beni-Suef University, Experimental Animals Ethical Committee (No. BSU/EAEC/PSAS/16/112018).

Rats were randomly divided into 3 groups (10 rats/group). The HgCl_2_ dose was 4 mg/kg, i.p.:

  Group I (control): rats received saline i.p. for 14 days and served as the control
  Group II (HgCl_2_): rats were injected with a single dose of HgCl_2_ (4 mg/kg, i.p.) at day one of the experiment
  Group III (HgCl_2_ + DPPD): rats were injected with a single dose of HgCl_2_ (4 mg/kg, i.p.) at day one of the experiments and, then, treated with DPPD (0.5 g/kg, i.p.) according to [15] once every two days starting from day 3 of the experiment

### 2.3. Collection and Preparation of Samples

All rats were exposed to sevoflurane anesthesia and killed by decapitation (24 h after the last injection), and urine and blood samples were collected from each rat after 14 days of HgCl_2_ (or saline) injection. The liver and kidneys tissues were dissected and used for biochemical, flow cytometry, and histopathological examinations.

### 2.4. Liver and Renal Markers

To assess liver functions, serum ALT, AST, and ALP enzyme activities were detected. Also, serum urea, uric acid, and creatinine levels were determined to assess kidney functions using colourimetric diagnostic kits (Biodiagnostic, Cairo, Egypt) according to the manufacturer's instructions.

### 2.5. Oxidative Stress Evaluation

The activities of the antioxidant enzymes SOD, CAT, and GSH in addition to the MDA content in liver and kidney tissues were all measured using commercial laboratory diagnostic kits (Biodiagnostic Co., Cairo, Egypt).

### 2.6. CD8%, CD4%, and TGF-*β*% Measurements

Flow cytometry detection of CD8%, CD4%, and TGF-*β*% depends on the specific binding of monoclonal antibodies to the antigenic determinants. The monoclonal antibodies labelled with different fluorochromes which are excited via a laser beam from a flow cytometer during analysis. The fluorescence intensity differences were proportional to the expression of the analyzed antigens. Assays were analyzed by using an Accuri C6 flow cytometer (BD Biosciences, San Jose, CA). Data were quantified with C Flow software (BD Accuri, San Jose, CA).

### 2.7. Measurement of the Hydroxyproline Content

About 50 mg of kidney or liver tissue specimens were hydrolyzed, and then, chloramine *T* solution was added to the specimen's supernatant and, then, incubated, followed by Ehrlich's solution addition. The final mixture was incubated, and the optical density was estimated at 560 nm [16]. Hydroxyproline values were expressed as ug/mg tissue.

### 2.8. Histopathological Examination

The formalin-embedded liver and kidney tissues were cut into 4 *μ*m thick sections, and then, the slides were stained with hematoxylin and eosin (H&E) for histological evaluation and Masson trichrome to assess collagen deposition. We routinely conduct H&E staining to grade tubular damage (0, no damage; 1, 0–25% damaged tubules; 2, 25–50% damaged tubules; 3, 50–75% damaged tubules; and 4, >75% damaged tubules) [17] and the liver injury score of fibrosis as described by [18]. The sections were examined and photographed using an Olympus light microscope (Olympus BX51, Tokyo, Japan) with an attached digital photograph machine (Olympus E-330). Images were captured from each section randomly, and semiquantitative analysis of the fibrotic area was performed on an Intel® Core I5®-based computer using Image *J* software with a specific built-in routine for stain quantification and automated area measurement. Five slides were prepared from each group, 5 random fields from each slide analyzed as previously reported [19, 20].

### 2.9. Statistical Analyses

Data were analyzed using SPSS software version 22 for Windows (IBM, Armonk, NY, USA). Descriptive statistics were calculated in the form of Mean ± Standard deviation (SD). ANOVA and Tukey's post hoc tests were used for comparison between groups. A level of *p* < 0.05 was defined as statistically significant.

## 3. Results

### 3.1. Liver and Kidney Functions

The potential effects of HgCl_2_ and DPPD treatment on renal and liver function parameters are summarized in Tables 1 and 2. The presented data showed that serum ALT, AST, ALP, creatinine, urea, and uric acid levels were significantly (*p* < 0.001) increased in HgCl_2_-injected rats compared to control and HgCl_2_ + DPPD-treated rats. Conversely, animals treated by HgCl_2_ + DPPD reversed all parameters' alterations towards the normal ranges.

### 3.2. Lipid Peroxidation and Antioxidant Enzyme Activities

The data of lipid peroxidation, CAT, GSH, and SOD activities in the renal and hepatic tissues are shown in Tables 1 and 2. Compared with control and HgCl_2_ + DPPD group values, the HgCl_2_ group showed a significantly (*p* < 0.001) increased MDA level and significantly decreased antioxidant enzymes (CAT, GSH, and SOD) activities. These results indicate that DPPD ameliorates the HgCl_2_-induced oxidative stress in the liver and kidney.

### 3.3. Hepatorenal Fibrosis Induced by HgCl_2_ in Rats

The hydroxyproline content is a specific marker for collagen deposition. The HgCl_2_ group showed a significantly (*p* < 0.001) increased hydroxyproline content in renal and liver tissues compared with the control group. In contrast, DPPD treatment significantly decreased the renal and liver hydroxyproline content (Tables 1 and 2).

### 3.4. Flow Cytometry

As shown in Tables 1 and 2, the TGF-*β*, CD4, and CD8 percent showed a significant (*p* < 0.001) increase in HgCl_2_-treated rats compared to control and HgCl_2_ + DPPD-treated rats. Conversely, animals treated by DPPD reversed TGF-*β*, CD4, and CD8 percent alterations towards the normal ranges.

### 3.5. Histopathological Analysis

**Figure 1:**
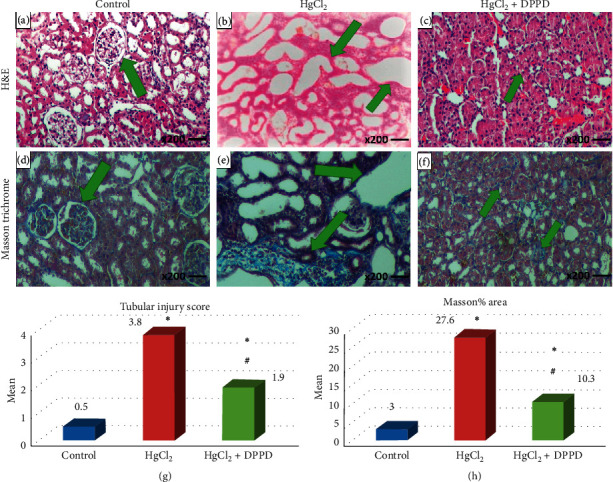
H&E-stained and Masson-stained kidney tissues of rats from different groups (magniﬁcation ×200). (a) Section of the control group showing the normal architecture of the kidney. (b) Significant increase in tubular dilatation and degenerative changes observed in HgCl_2_-injured rats. (c) Treatment with DPPD significantly attenuated the renal histopathological changes. (d) Masson's trichrome staining indicated no abnormal collagen in the control group. (e) Sections of HgCl_2_-treated group indicated an increase in fibrosis stained in blue. (f) Kidney section of rats cotreated with HgCl_2_ + DPPD showed a significant decrease in collagen deposits. (g) Pathological scoring showed a significant increase in the tubular injury score in the HgCl_2_-treated group when compared with other groups. (h) Comparison between different groups in the Masson% area. Data were mean ± SD. ^*∗*^*p* < 0.01 vs. control; ^#^*p* < 0.01 vs. HgCl_2_.

**Figure 2:**
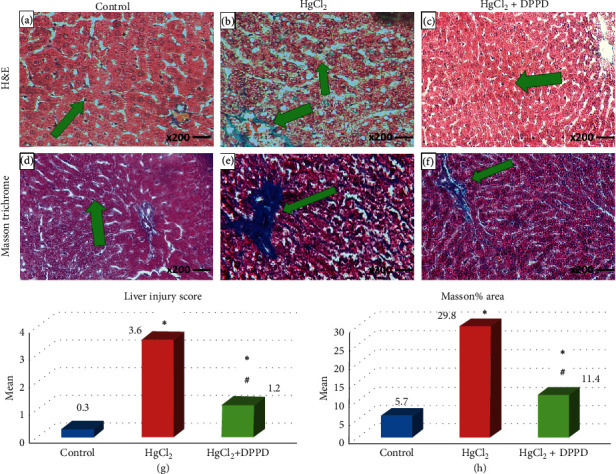
H&E-stained and Masson-stained liver tissues of rats from different groups (magnification ×200). (a) Hepatic histology of the control group, showing normal hepatic lobular architecture. (b) Hepatic degenerative changes with extensive cell necrosis observed in HgCl_2_-injured rats. (c) Rats treated with DPPD showed a significant modulation in the hepatic histology towards normal. (d) Control group stained with Masson's trichrome showed that the natural structure and collagen fibers cannot be seen. (e) Accumulation and progression of collagen fibers in the liver of the HgCl_2_ group. (f) Significant decrease in collagen fibers observed in rats cotreated with HgCl_2_ + DPPD. (g) Pathological scoring showed a significant increase in the hepatic injury score in the HgCl_2_-treated group compared with other groups. (h) Comparison between different groups in the Masson% area. Data were mean ± SD. ^*∗*^*p* < 0.01 vs. control; ^#^*p* < 0.01 vs. HgCl_2_.

H&E-stained and Masson trichrome-stained kidney sections are shown in Figure 1 and liver sections are shown in Figure 2. H&E histopathological stain in the control group showed normal kidney morphology (Figure 1(a)) and normal hepatic lobular architecture with distinct hepatocytes (Figure 2(a). HgCl_2_-treated animals showed tubular dilatation with many degenerated signs (Figure 1(b)) and hepatic degeneration with large areas of extensive cell necrosis (Figure 2(b)). Treatment with DPPD significantly attenuated the pathological changes in both kidney (Figure 1(c)) and liver (Figure 2(c)) tissues compared to the HgCl_2_-treated group. Assessment of kidney (Figure 1(g)) and liver (Figure 2(g)) injury by a semiquantitative scoring system from 0 to 5. Data were mean ± SD. ^*∗*^*p* < 0.01 vs. control, ^#^*p* < 0.01 vs. HgCl_2_.

The collagen content was assessed by Masson's trichrome stain, and the control group showed a normal collagen content in the kidney (Figure 1(d)) and liver (Figure 2(d)) tissues. Significant amounts of collagen deposition were observed in the kidney (Figure 1(e)) and liver (Figure 2(e)) tissues of HgCl_2_-treated animals. Conversely, oadministration of DPPD + HgCl_2_ showed a significant (*p* < 0.001) modulation in the collagen content level towards normal in both renal (Figure 1(f)) and hepatic (Figure 2(f)) tissues. Treatment with DPPD significantly (*p* < 0.001) decreased both the renal (Figure 1(h)) and hepatic (Figure 2(h)) Masson% area.

## 4. Discussion

HgCl_2_ generates free radicals and subsequently increases oxidative stress, which leads to nephrotoxicity and accelerates hepatotoxicity [21]. This adverse effect of HgCl_2_ could be eliminated by DPPD treatment probably because of its strong free radical scavenging activity through the electron(s) donation pathway, protecting cells from oxidation and necrosis [11].

In the present study, liver and renal functions were detrimentally altered after HgCl_2_ administration causing hepatorenal dysfunction evidenced by a significant elevation in AST, ALT, and ALP enzyme activities and urea, uric acid, and creatinine levels. Similar results were reported by [22, 23]. Treatment with DPPD showed a marked improvement that was clear in the previously listed parameters, and these findings agreed with those of Kawai et al. [13] who reported that DPPD may possess an antioxidative behavior.

HgCl_2_ administration initiates the formation of highly reactive substances such as reactive oxygen species in addition to the stimulation of oxidative stress [24]. Consequently, the lipid peroxidation level increased and the antioxidant enzymes activities decreased.

In the present study, we found that HgCl_2_ significantly diminishes the activities of the antioxidant enzymes SOD and CAT in addition to GSH in kidney and liver tissues, whereas the end product of lipid peroxidation (MDA contents) was significantly increased compared with the control group. A variety of experiments have demonstrated parallel results [23, 25]. Conversely, coadministration of DPPD + HgCl_2_ showed a significant modulation in the activities of SOD and CAT in addition to the level of GSH and MDA towards normal. The hepatorenal protective activity of DPPD was observed in the previous studies [12, 26].

Hydroxyproline is used for the estimation of the collagen content, considering that collagen contained 12.7% hydroxyproline by weight [27]. Our results were parallel to those of Yuan et al. [28] who concluded that renal and liver fibrosis are induced by HgCl_2_, demonstrated by a significant elevation (*p* ≤ 0.001) of the hydroxyproline content in liver and kidney tissues compared to control. Consequently, treatment with DPPD significantly attenuated hydroxyproline and collagen deposition (*p* ≤ 0.001) in animals [19].

Our results reported a significant increase in both renal and hepatic TGF-*β*%, CD4%, and CD8% in HgCl_2_-treated rats compared to (control and HgCl_2_ + DPPD groups). The elevated CD4+ and CD8+ percentage may be related to the abnormal immune status due to HgCl_2_ toxicity. These results are in agreement with the previous findings by Liu et al. [29], who indicate significant increases in CD4% and CD8% as a result of mercury induction of T-cell autoimmune syndrome, including autoantibodies and increases in TGF-*β* production and various other cytokines cause collagen deposition. Moreover, previous studies [30, 31] reported a significant increase in renal and hepatic TGF-*β*% in HgCl_2_-treated rats compared to control, but rats treated with HgCl_2_ + DPPD showed a significant decrease in TGF-*β*%, CD4%, and CD8% towards normal.

In the present study, we performed a histopathological examination to further support the biochemical and immunological evidence. We compared the morphological structure among each group using H&E stain. In the control group, there were no injuries or histological changes detected in the kidneys (Figure 1(a)) or liver (Figure 2(a)). The HgCl_2_ group showed liver necrosis, swelling, and structure changes (Figure 2(b)) in addition to renal tissue damage, collagen formation, and atrophy in the normal tubular architecture (Figure 1(b)) compared with (control and HgCl_2_ + DPPD groups). Treatment with DPPD significantly attenuated the pathological changes in both kidney (Figure 1(c)) and liver (Figure 2(c)) tissues. Earlier studies [4, 32] demonstrated parallel results.

Masson's trichrome stain was used for distinguishing collagen deposition. The control group showed a normal collagen content in the kidney (Figure 1(d)) and liver (Figure 2(d)) tissues, while in the HgCl_2_ group, the collagen content was increased significantly in the kidney (Figure 1(e)) and liver (Figure 2(e)). Our results agreed with those in [33, 34]. The recorded hepatorenal lesions and collagen content in HgCl_2_ + DPPD cotreated rats decreased significantly (*p* < 0.001) compared with HgCl_2_-treated rats (Figure 1(f)) and (Figure 2(f)). Similar protective effects for DPPD were previously reported in [13].

## 5. Conclusions

Finally, we conclude that the antioxidant DPPD can retard the progression of hepatorenal fibrosis and collagen deposition induced by HgCl_2_. Further studies are needed to explain the intrinsic and extrinsic pathways of DPPD antifibrotic efficacy.

## Figures and Tables

**Table 1:** Kidney injury parameters. Values are expressed as *M* ± SD of 10 animals in each group.

Variables	Control group	HgCl_2_ group	HgCl_2_ + DPPD group
Serum:			
Creatinine (mg/dl)	0.46 ± 0.03	1.53 ± 0.21^*a*^	0.57 ± 0.10^*b*^
Urea (mg/dl)	27.83 ± 3.60	73.43 ± 5.87^*a*^	32.23 ± 2.30^*b*^
Uric acid (mg/dI)	2.57 ± 0.25	4.53 ± 0.76^*a*^	3.16 ± 0.98^*a*,*b*^

Homogenate:			
Hydroxyproline (ug/mg tissue)	22.58 ± 0.63	41.23 ± 9.25^*a*^	30.24 ± 5.23^*a*,*b*^
MDA (mmol/g tissue)	59.79 ± 4.99	92.25 ± 13.05^*a*^	70.62 ± 9.69^*b*^
SOD (U/mg protein)	11.66 ± 0.64	6.71 ± 1.51^*a*^	9.63 ± 1.48^*a*,*b*^
CAT (mol/min/gm)	0.66 ± 0.09	0.35 ± 0.05^*a*^	0.58 ± 0.09^*b*^
Glutathione (*μ*mol/g protein)	32.13 ± 2.07	21.71 ± 6.22^*a*^	28.21 ± 3.51^*b*^
TGF-*β* (ng/ml)	33.27 ± 3.43	49.20 ± 9.10^*a*^	38.60 ± 9.19^*b*^
CD4 (ng/ml)	21.42 ± 1.04	43.70 ± 5.31^*a*^	34.30 ± 5.01^*a*,*b*^
CD8 (ng/ml)	23.62 ± 3.88	44.06 ± 6.77^*a*^	25.66 ± 3.87^*b*^

SD: standard deviation; P : probability; ^*∗*^significance <0.05; ^*∗∗*^high significance. The test used is one-way ANOVA followed by post hoc Tukey. ^*a*^Significance relative to the control group compared with HgCl_2_ and HgCl_2_ + DPPD groups. ^*b*^Significance between the HgCl_2_ group and HgCl_2_ + DPPD group.

**Table 2:** Liver injury parameters. Values are expressed as *M* ± SD of 10 animals in each group.

Variables	Control group	HgCl_2_ group	HgCl_2_ + DPPD group
Serum:			
ALT (U/L)	33.30 ± 3.30	64.70 ± 14.16^*a*^	41.80 ± 11.14^*b*^
AST (U/L)	59.40 ± 5.12	114.70 ± 15.96^*a*^	74.00 ± 5.43^*a*,*b*^
ALP (U/L)	215.45 ± 5.42	333.97 ± 32.48^*a*^	247.10 ± 25.91^*a*,*b*^

Homogenate:			
Hydroxyproline (ug/mg tissue)	19.80 ± 1.84	39.20 ± 1.77^*a*^	24.36 ± 4.08^*a*,*b*^
MDA (mmol/g tissue)	81.83 ± 4.58	112.72 ± 10.48^*a*^	81.93 ± 4.02^*b*^
SOD (U/mg protein)	20.99 ± 1.75	14.87 ± 2.65^*a*^	19.94 ± 3.71^*b*^
CAT (mol/min/gm)	1.05 ± 0.10	0.60 ± 0.28^*a*^	0.93 ± 0.13^*b*^
Glutathione (*μ*mol/g protein)	25.35 ± 1.50	18.97 ± 3.10^*a*^	22.24 ± 2.01^*a*,*b*^
TGF-*β* (ng/ml)	32.31 ± 2.45	50.60 ± 8.83^*a*^	34.60 ± 4.92^*b*^
CD4 (ng/ml)	24.44 ± 2.64	39.66 ± 1.60^*a*^	25.30 ± 3.31^*b*^
CD8 (ng/ml)	19.73 ± 1.13	38.5 ± 2.88^*a*^	21.07 ± 3.84^*b*^

SD: standard deviation; P : probability; ^*∗*^significance <0.05; ^*∗∗*^high significance. The test used was one-way ANOVA followed by post hoc Tukey. ^*a*^Significance relative to the control group compared with HgCl_2_ and HgCl_2_ + DPPD groups. ^*b*^Significance between the HgCl_2_ group and HgCl_2_ + DPPD group.

## Data Availability

The data used to support the findings of this study are available from the corresponding author upon request.
